# Brain Development in Children With Early Onset Liver Disease

**DOI:** 10.1192/bjo.2022.81

**Published:** 2022-06-20

**Authors:** Megan Earl, Charlotte Blackmore, Jemma Day, Marianne Samyn, Anil Dhawan, Grainne McAlonan

**Affiliations:** 1 Kings College London, London, United Kingdom; 2 Kings College Hospital, London, United Kingdom

## Abstract

**Aims:**

Biliary Atresia (BA) is a progressive inflammatory liver disease and the most frequent indication for pediatric liver transplant. There is a strong association between BA in adulthood and reduced cognitive abilities, however, data on neurodevelopmental outcomes at an early age are scarce, with small participant numbers. Hence, the neurodevelopmental difficulties in BA are poorly understood in childhood even though the brain development and maturation occur in parallel with the time-course of BA. This study aimed to characterize the cognitive and behavioral phenotype within BA infants from the age of 14 months to 4 years and investigate the extent to which this group deviates from children of typical development.

**Methods:**

42 infants with BA that were diagnosed and treated at Kings College Hospital were recruited into this study. These infants ranged from 14 months to 4 years (mean age = 3 Years, 1 month). Out of the 42 infants, 19 had received a liver transplant, 22 were stable on their native liver, and 1 was on the transplant waiting list. 36 Mullens Scale of Early Learning assessments and 42 Vineland Adaptive Behavior Scale Interviews were collected. 42 typically developing infants (TD) were also recruited into the study, matched for age and gender to the BA population. First, we compared the whole group with BA to TD; then we compared children with BA on their native liver to those with a transplant.

**Results:**

Across the cohort with BA, infants scored significantly lower on the Vineland Summary T-Score compared to age-matched TD control children (t(82) = −5.05, p < .001) and across all domains of the Vineland. They also scored significantly lower than TD children on the Mullens Development Assessment (t(66) = −6.52, p < .001), and this was also across all domains. BA children on their native liver scored lower on both instruments than children who had received a liver transplant, however, this difference did not reach significance.

**Conclusion:**

Individuals with Biliary Atresia, regardless of their transplant status, show lower levels of development across all aspects, suggesting a global delay. These findings suggest that all of these young children remain at significant risk for neurodevelopmental difficulties. These findings emphasize that special attention to neurodevelopment needs to be given as part of a holistic approach to care in a serious life-long illness. Work is ongoing to understand the trajectory of brain maturation in these children to ensure neurodevelopmental needs are addressed alongside physical health.

